# Phage Henu12-resistant mutant derives fitness trade-offs in *Shigella dysenteriae*

**DOI:** 10.1128/spectrum.01150-25

**Published:** 2025-10-27

**Authors:** Salwa E. Gomaa, Yuhan Wang, Jingjing Li, Huiru Cao, Jiawen Shen, Xiaotao Dong, Yangyang Liu, Na Chen, Qiming Li, Zhongyi Yan, Yulu Du, Xinying Ji, Jing Zhao, Yinqiong Tu, Tieshan Teng

**Affiliations:** 1 Henan International Joint Laboratory for Nuclear Protein Regulation, School of Basic Medical Sciences, Henan University, Kaifeng, Henan, China; 2 Department of Microbiology and Immunology, Faculty of Pharmacy, Zagazig University 68799, Zagazig, Egypt; 3 Department of Pulmonary and Critical Care Medicine, Huaihe Hospital, Henan University, Kaifeng, Henan, China; 4 Henan Provincial Research Center of Engineering and Technology for Medical Detection of Nuclear Protein, Zhengzhou Health College, Zhengzhou, Henan, China; 5 Department of Medicine, The People’s Hospital of Hua Country, Huaxian, Henan, China; CEB - Centre of Biological, Braga, Portugal

**Keywords:** *Shigella dysenteriae*, antimicrobial resistance, phage, synergy, phage resistant, fitness trade-offs

## Abstract

The rapid emergence of multidrug-resistant *Shigella dysenteriae* strains necessitates new therapeutic approaches, such as phage therapy. Phages are considered a promising adjunct to antibiotics. The rapid emergence of phage-resistant bacterial mutants represents a major challenge in clinical practice. Nevertheless, this sometimes comes with fitness trade-offs. Thus, understanding the fitness trade-offs is crucial prior to phage clinical application. In the present study, a new lytic *S. dysenteriae* phage, Henu12, was isolated. Henu12 was assigned to the *Caudoviricetes* class and demonstrated good thermal, pH, and chloroform stability. The phage genome comprises a 69,779 bp linear double-stranded DNA with 90 open reading frames and has the highest similarity with *Shigella* phage Sf15 and *Escherichia* phage vB_EcoM_3HA14. Interestingly, the genome did not contain integrases, virulence, or antibiotic resistance genes. A time-kill assay demonstrated synergistic effects with polymyxin B, tetracycline, and ceftazidime. The phage-resistant mutant demonstrated impaired adsorption, downregulation of the tested lipopolysaccharide biosynthetic genes, enhanced sensitivity to polymyxin B and tetracycline, and environmental stressors, impaired biofilm, and diminished *in vivo* colonization compared to the wild-type strain. The whole-genome resequencing of the phage-resistant mutant identified a multi-base insertion in the *waaH* gene involved in the lipopolysaccharide biosynthesis pathway, potentially responsible for the phage resistance phenotype, along with other mutations related to the associated trade-offs. Together, these findings provide new insights into the evolutionary interactions between phage resistance and bacterial fitness that could potentially offer a novel approach to treat resistant *Shigella dysenteriae* infections.

**IMPORTANCE:**

Phage therapy is a promising approach to combat the rise of multidrug-resistant bacteria. The emergence of phage-resistant bacterial strains is a potential complication associated with the application of phages. In the current study, we demonstrated that the synergistic use of phage Henu12 in conjunction with antibiotics not only enhances the bactericidal efficacy but also postpones the development of resistant strains. In addition, we provide a better understanding of the fitness trade-off that might fill some gaps in the future success of phage therapies in combating resistant shigellosis in clinical settings.

## INTRODUCTION

*Shigella* species are one of the most prevalent causes of diarrheal infection, known as shigellosis, and mostly affect children under the age of 5 (1). *Shigella* spp. are primarily transmitted via the fecal–oral route, with infection occurring at a very low dose of bacteria (10–500 cells). The outbreaks of *Shigella* spp. infection are mainly due to the consumption of contaminated water and food, malnutrition, poor hygiene, and lack of awareness (2, 3). The risk of multidrug-resistant (MDR) shigellosis is often associated with international travelers, homeless people, immunocompromised persons, and men who have sex with men (4). Recently, the incidence of MDR *Shigella* spp. has been rising in North American and Asian countries owing to climate change driven by the accelerated industrial growth. Climate change is a global concern, disrupting microbial ecosystems, weakening healthcare systems, and accelerating the spread of MDR pathogens, including *Shigella* spp. Moreover, interactions between genetic and environmental risk factors have been reported to threaten human health and contribute to disease (5, 6). *Shigella* spp. annually account for ~140 million cases and 600,000 deaths worldwide attributable to MDR strains (2). Of all *Shigella* serotypes, *Shigella dysenteriae* type 1 has been associated with the most severe cases of dysentery and high mortality rates (7).

While antibiotics are usually prescribed for treating shigellosis, their indiscriminate use has resulted in the emergence of multidrug-resistant *Shigella* spp., which undermines the successful control of the disease and poses a significant public health threat (8). Therefore, the exploration of alternative therapeutic approaches is urgently required to combat the rising antimicrobial resistance. Phage therapy has garnered increasing attention for treating bacterial infections. Phage therapy has been reported as an effective adjunct to the existing antibiotics (9, 10). Shigellosis is considered one of the earliest human diseases successfully treated with phages (11). Subsequently, attempts at phage therapy were expanded to treat other bacterial infections such as cholera, conjunctivitis, and skin infections (12). However, with the interplay between bacteria and phages, the emergence of phage-resistant bacterial mutants is inevitable and is considered a major concern regarding the clinical application of phages. Several studies reported the development of phage-resistant bacteria *in vitro* and in humans (13, 14). Bacteria evolve resistance to phages through several mechanisms, among which are the prevention of phage adsorption through mutations in phage receptor sites, restriction-modification, the CRISPR/Cas system, and the abortive infection (15).

On the other hand, phage resistance has been recently reported to cause fitness costs in *S. flexneri*, manifested as attenuated virulence and increased sensitivity to certain antibiotics (16). Additionally, phage-resistant bacteria tend to become more vulnerable to the host-clearance mechanisms (17). However, such fitness costs may vary among different bacterial genera and species (18, 19). Accordingly, a better understanding of the potential of the fitness trade-offs as a countermeasure against phage resistance is crucial prior to phage clinical application. The current study aimed to isolate a novel lytic phage, Henu12, investigate its biological and genomic characteristics, evaluate the therapeutic effectiveness of certain phage-antibiotic combinations against *S. dysenteriae,* and assess whether the development of phage-resistant mutants might result in fitness trade-offs that could provide a new strategy for combating resistant *shigellosis*.

## RESULTS

### Morphological characterization of Henu12

**Fig 1:**
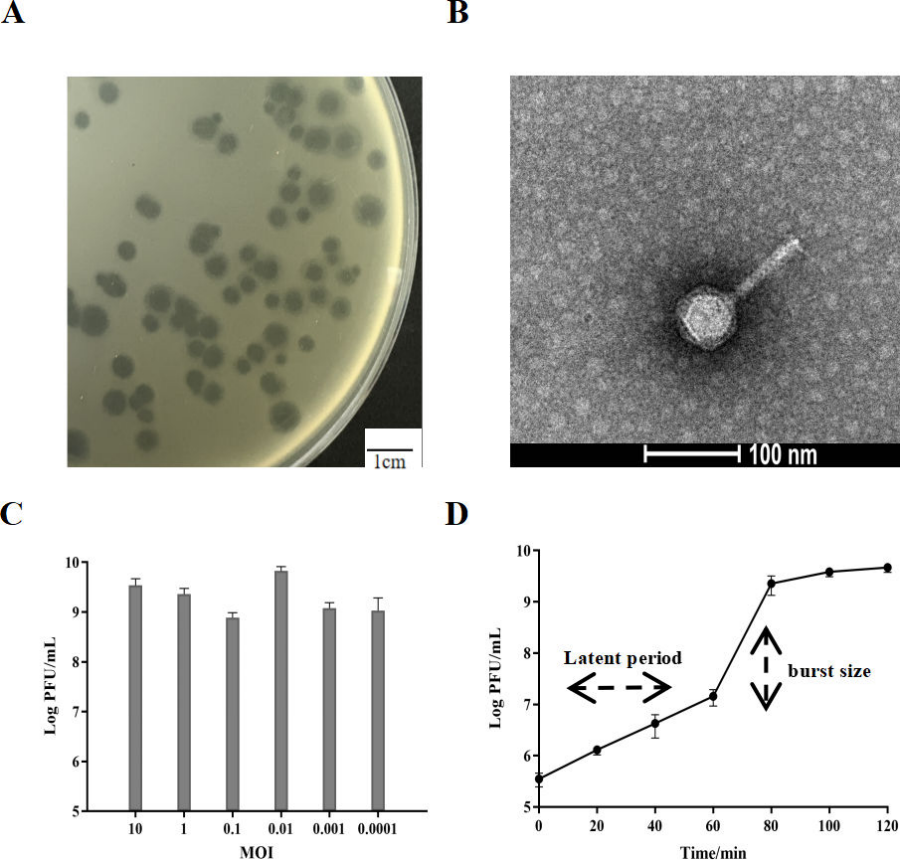
Characterization of *S. dysenteriae* phage Henu12. (**A**) The plaque morphology. (**B**) Electron micrograph of phage particles. (**C**) The optimal multiplicity of infection (MOI). (**D**) The one-step growth curve at MOI (0.01).

A novel lytic phage, named Henu12, was isolated from sewage samples collected from a poultry farm using *S. dysenteriae* as the host. Phage Henu12 produced distinct round, clear plaques on the bacterial host lawn surrounded by translucent halo zones (0.5–1 mm) in diameter (Fig. 1A). TEM showed that phage Henu12 has a contractile tail of about 169 nm long and an icosahedral head (44 ± 1 nm in diameter) (Fig. 1B). According to the ICTV guidelines, phage Henu12 has been assigned to the *Caudoviricetes* class.

### Biological characteristics of phage Henu12

The highest progeny phage titer of 8.4 × 10^9^ PFU/mL was demonstrated at a multiplicity of infection (MOI) equal to 0.01, indicating that this was the optimal MOI for phage Henu12 (Fig. 1C). The one-step growth curve showed that phage Henu12 had a latent period of about 60 min and an average burst size of 50 PFU/cell (Fig. 1D).

### Phage Henu12 stability

**Fig 2:**
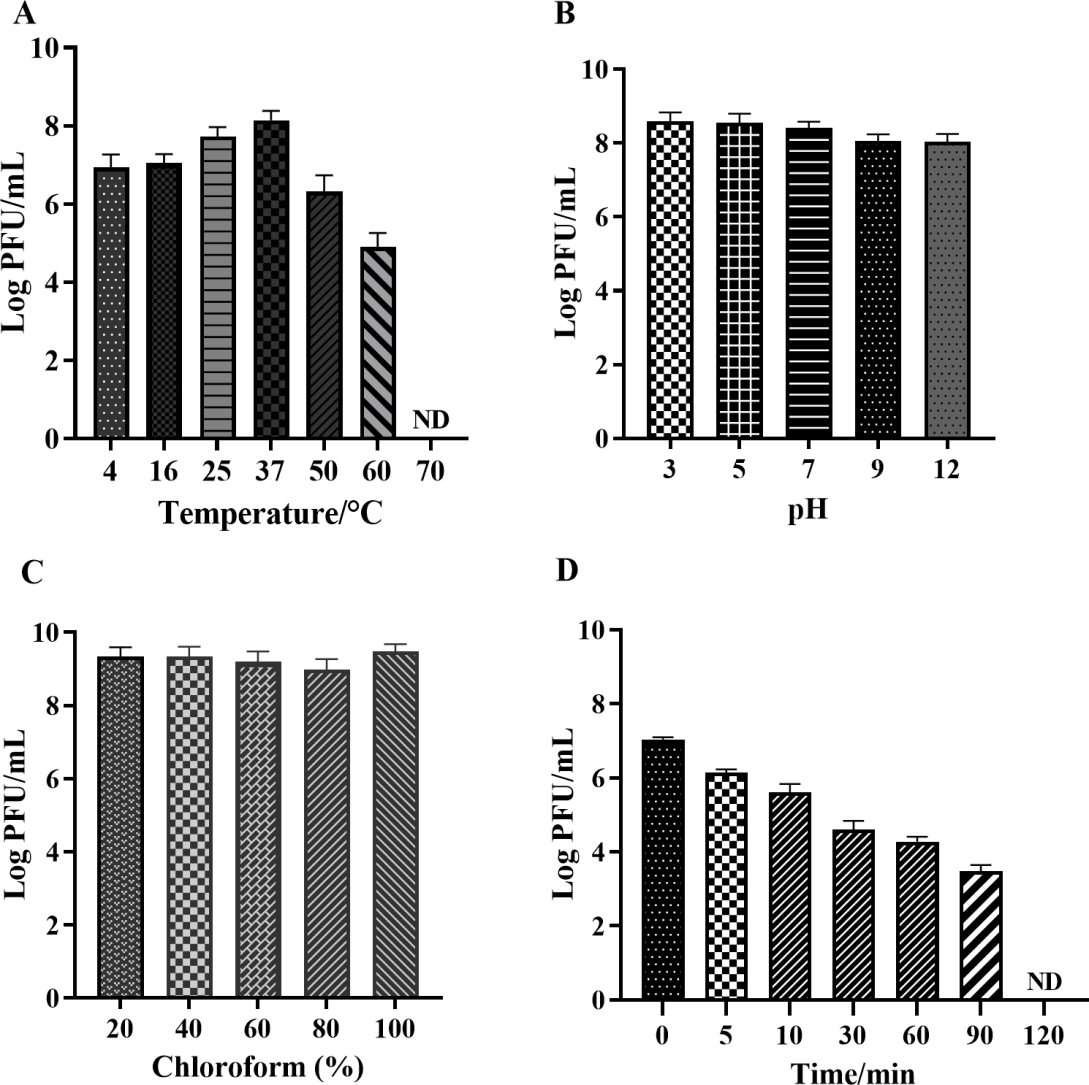
Stability of phage Henu12. (**A**) Phage Henu12 was thermally stable between 4°C and 37°C. (**B**) Phage Henu12 endured varying pH levels (3–12). (**C**) Phage Henu12 was stable against chloroform. (**D**) Phage Henu12 lost its infectivity after 90 min of UV irradiation. ND, not detected.

The stability of phage Henu12 against various temperatures, pH ranges, and chloroform concentrations was assessed after 1-h incubation. The thermal stability test showed that phage Henu12 endured temperatures up to 37°C; however, a noticeable reduction in phage infectivity was demonstrated at 50°C and 60°C with complete loss at 70°C (Fig. 2A). In addition, phage Henu12 could survive in wide pH ranges (3–12) (Fig. 2B). Moreover, phage Henu12 retained its activity after chloroform treatments (Fig. 2C). Regarding phage Henu12 sensitivity to UV irradiation, the phage titer gradually reduced over time before being dropped to zero after 90 min, as shown in Fig. 2D. Altogether, these findings underscore the potential of phage Henu12 for therapeutic applications.

### Genome annotation and bioinformatics analysis

**Fig 3:**
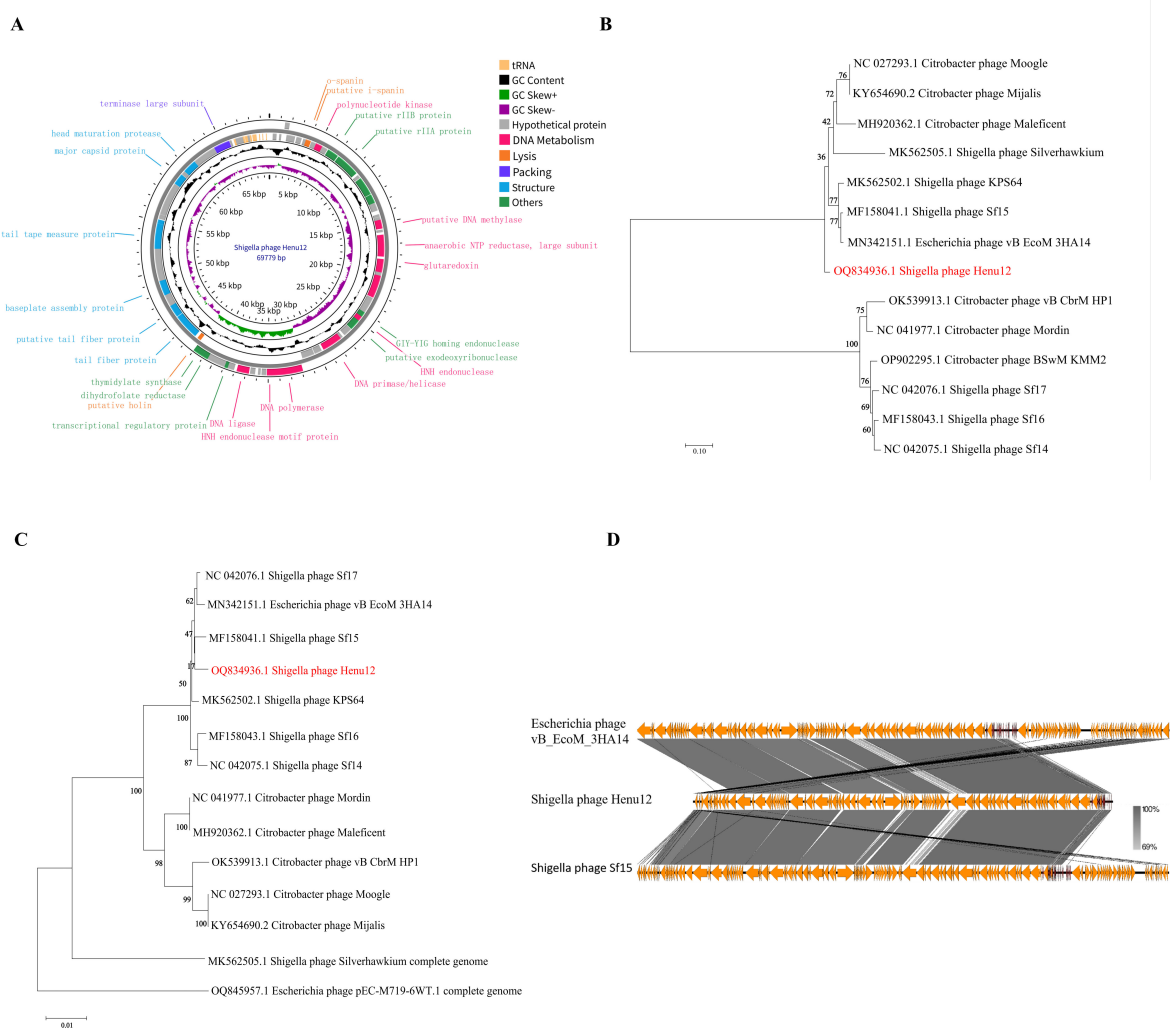
Genomic features of phage Henu12. (**A**) Circular genomic map of phage Henu12. The inner rings represent genome location, GC skew positive (green), GC skew negative (purple), and GC content (black). Functional ORFs are classified into four groups: nucleic acid metabolism-related proteins (pink), lysis-related proteins (dark orange), packaging-related proteins (dark blue), and structural proteins (light blue). Hypothetical proteins are shown in gray. The figure was generated using the CGView program (https://proksee.ca/). (**B**) Phylogenetic analysis of phage Henu12 and 13 other closely related sequences. (**C**) Phylogenetic analysis based on the amino acid sequences of large subunit terminase. Phylogenetic trees were constructed using MEGA11 software using the neighbor-joining method. Phage Henu12 was colored red. (**D**) Pairwise BLAST comparison of phage Henu12 with homologous phages (*Escherichia* phage vB_EcoM_3HA14 and *Shigella* phage Sf15) using Easyfig. Similarity level among phage sequences is represented by the colored scale bar from 69% to 100%. The coding sequences are represented by directional arrows.

Phage Henu12 has a linear double-stranded DNA genome (69,779 bp long), with a G + C content of 39.40% and a total of 90 open reading frames (ORFs): 18 are located in the positive strand and 72 ORFs in the negative strand. The annotation results identified that 56 of these ORFs encode hypothetical proteins, whereas the remaining 34 ORFs produce functional proteins. The 34 annotated functional proteins are categorized into four module groups, including DNA metabolism, lysis, packaging, and structure. Specifically, there are 12 genes related to DNA metabolism, 3 genes associated with lysis, 7 genes linked to the structural module, 1 gene connected to the packaging module, and 11 genes with other functions (Fig. 3A; Table S2). Interestingly, a cluster of 18 tRNA genes was predicted in the Henu12 phage genome (Table S3). Additionally, taxMyPhage analysis predicted phage Henu12 to be a new species of the genus *Mooglevirus* of the *Caudoviricetes* class. Notably, none of the ORFs encoded antibiotic resistance, integrases, or bacterial virulence factors, which support its potential use in clinical settings.

Phylogenetic analysis using the complete genome sequence of 13 other related phages retrieved from the GenBank showed that phage Henu12 shares the highest similarity with two other Moogleviruses: *Shigella* phage Sf15 (MF158041.1) and *Escherichia* phage vB_EcoM_3HA14 (MN342151.1), representing percent identities of 97.11% and 93%, respectively (Fig. 3B). Additionally, a phylogenetic tree was constructed based on the large terminase subunit to further elucidate the evolutionary relationships between Henu12 phage and other phages. The results showed a high degree of similarity, consistent with the whole-genome phylogeny (Fig. 3C). Genome comparison using Easyfig software demonstrated strong homology among Henu12 phage, *Shigella* phage Sf15, and *Escherichia* phage vB_EcoM_3HA14, with only minor genetic variations. These differences were primarily localized within the structural and functional modules (Fig. 3D).

### Phage-antibiotic combination is better than either phage or antibiotic alone

Prior to determining the possible synergistic interactions between phage Henu12 and six different antibiotics (polymyxin B [POL], tetracycline [TCY], ceftazidime [CAZ], ciprofloxacin [CIP], ampicillin [AMP], and rifampicin [RIF]), the MIC values for each antibiotic are represented in Table 1. Afterward, a time-kill assay was conducted to assess the potential synergy between the phage Henu12 (MOI = 0.01) and 1/2 MIC values of these antibiotics. Interestingly, the phage-POL combination significantly inhibited the emergence of phage-resistant strains. While phage-CAZ and phage-CIP combinations delayed the emergence of resistance, the combined action was insufficient to completely suppress it. Moreover, combining TCY, AMP, or RIF with phage Henu12 neither inhibited nor delayed the emergence of resistance.

**TABLE 1:** Minimum inhibitory concentrations of various antibiotics

Antibiotic class	Antibiotics	MIC (µg/mL)
Quinolones	Ciprofloxacin	0.05
Polymyxins	Polymyxin B	8
Tetracyclines	Tetracycline	10
Rifamycins	Rifampicin	5
Beta-lactams	Ceftazidime	0.6
	Ampicillin	30

**Fig 4:**
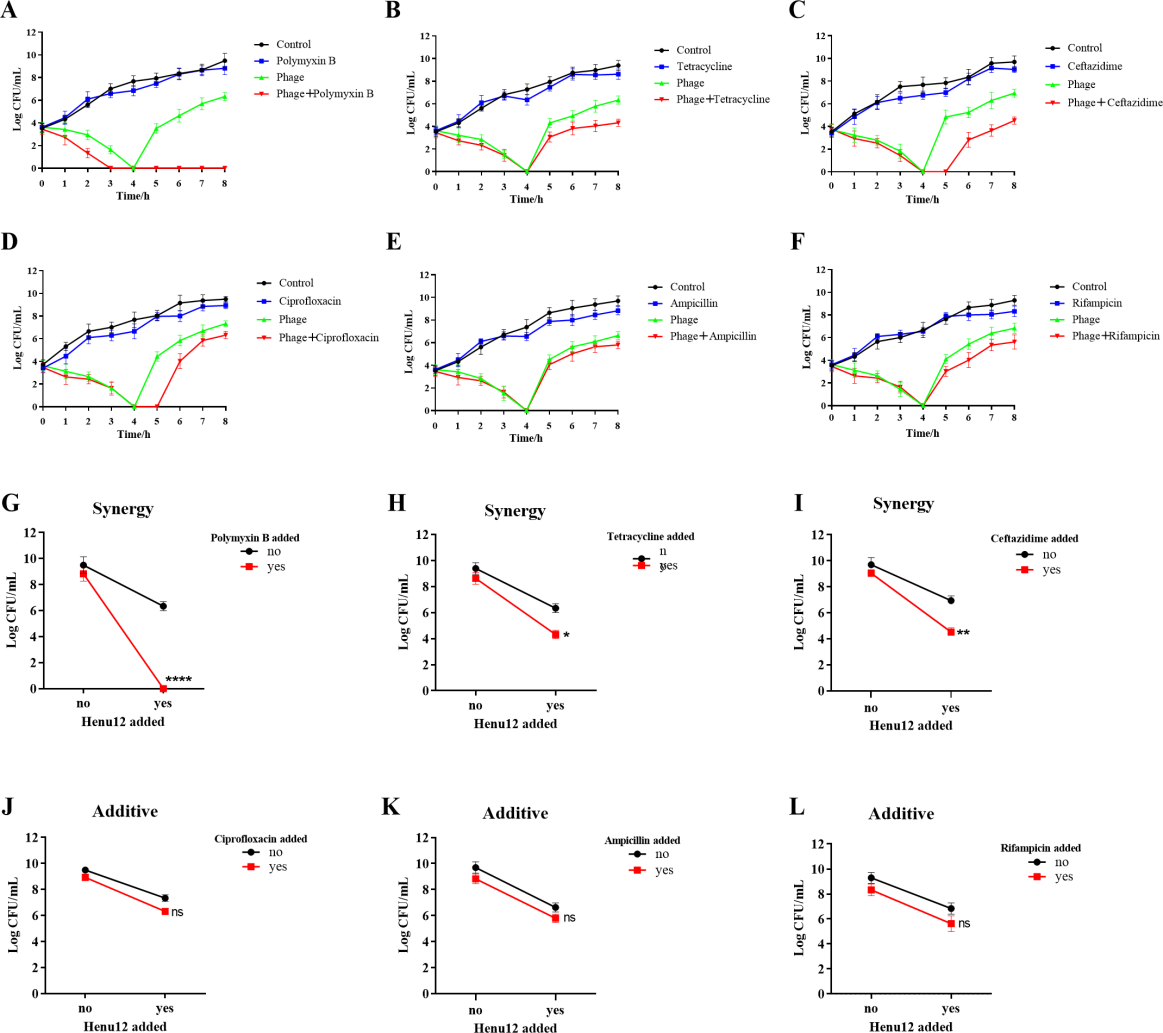
Time-kill analysis and interaction plots for phage-antibiotic synergy. Bacterial growth was assessed over 8 h in the presence or absence of phage Henu12 and antibiotic (**A–F**), and synergy was investigated via interaction plots (**G–L**). (**A**) Polymyxin B. (**B**) Tetracycline. (**C**) Ceftazidime. (**D**) Ciprofloxacin. (**E**) Ampicillin. (**F**) Rifampicin. (**G**) Polymyxin B. (**H**) Tetracycline. (**I**) Ceftazidime. (**J**) Ciprofloxacin. (**K**) Ampicillin. (**L**) Rifampicin. Two-way ANOVA was employed to analyze these interaction plots for statistical significance. *P < 0.05, **P < 0.01, and ****P < 0.0001.

The use of phage Henu12 with POL in combination led to a reduction in bacterial load exceeding 99%. Notably, no detectable bacterial counts were found in the phage-POL combination after 2 h of treatment. The phage-POL combination treatment was 4.6-fold more effective than POL alone and 2.2-fold more effective than the phage alone (Fig. 4A). The bactericidal activities of combining TCY or CAZ with phage Henu12 were also enhanced compared to individual treatments (Fig. 4B and C). The efficacy of either phage-TCY or the phage-CAZ combinational treatments was two times higher than that of either antibiotic alone and 1.5 times higher than that of the phage alone. The bactericidal effectiveness of combining phage-CIP, phage-AMP, or phage-RIF was approximately equal to the sum of their individual effects (Fig. 4D, E, and F). Through the interaction plots, POL, TCY, and CAZ were determined to act synergistically with phage Henu12, but additive effects were shown with the other antibiotics (Fig. 4G through L).

### Confirmation of phage-resistant phenotype

**Fig 5:**
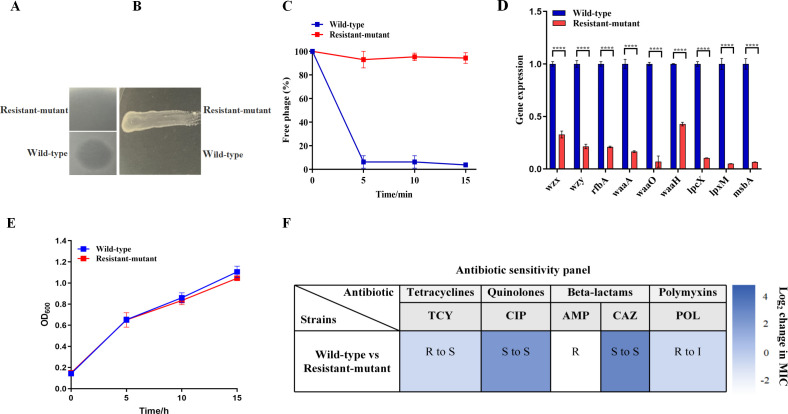
Phenotypic characterization of the phage-resistant *S. dysenteriae* mutant. (**A**) Spot assay of phage Henu12 when plated on the wild type and the phage-resistant mutant. (**B**) The inverted spot assay containing phage Henu12. (**C**) Phage adsorption assay on the wild type and the phage-resistant mutant. (**D**) Relative expression for genes associated with lipopolysaccharide biosynthesis in the wild type and the phage-resistant mutant was detected using quantitative reverse transcription PCR. (**E**) Growth curve of the wild type and the phage-resistant mutant. (**F**) Sensitivity of the wild type and the phage-resistant mutant to different antibiotics: tetracycline, ciprofloxacin, ampicillin, ceftazidime, and polymyxin B. Sensitivity is classified as S (sensitive), I (intermediate), or R (resistant). ****P < 0.0001.

After co-culturing phage Henu12 with the *S. dysenteriae* host strain for 48 h, the frequency of phage*-*resistant mutants was calculated for the emerging colonies, demonstrating a mutation frequency of 1.51 × 10^−7^. Subsequently, one phage-resistant mutant was picked and verified for the phage resistance phenotype. The results of the spot assay showed that phage Henu12 formed clear plaques when plated on the wild-type strain. Conversely, no lytic activity was noted with the phage-resistant mutant (Fig. 5A). On the inverted spot agar plate, the phage-resistant mutant experienced confluent growth, as opposed to no growth with the wild-type strain (Fig. 5B). When grown on LB agar, the wild type and its phage-resistant mutant produced phenotypically similar colonies.

### Adsorption assay

The adsorption assay demonstrated that approximately 96% of the phage Henu12 particles were able to adsorb to the wild-type strain within 15 min. The adsorption rate constant was calculated to be 2.19 × 10^−9^ mL/min (Table S4). By contrast, no phage adsorption was noticed with the phage-resistant mutant (Fig. 5C).

### The phage-resistant mutant exhibited altered LPS gene expression

The quantitative reverse transcription PCR (qRT-PCR) showed that the lipopolysaccharide (LPS)-related gene (*wzx, wzy, rfbA, waaA, waaO, waaH, lpcX, lpxM, *and *msbA*) expression was markedly reduced in the phage-resistant mutant compared to that of the wild-type strain. The expression levels of the aforementioned genes in the phage-resistant mutant decreased significantly by approximately 3-, 4.6-, 4.7-, 6-, 14.2-, 2.3-, 9.6-, 19.6-, and 15.2-fold, respectively, when compared to the wild-type strain (*P* < 0.0001). This could be responsible for the impaired adsorption noted with the phage-resistant mutant, suggesting the potential role of LPS as a receptor for phage Henu12 (Fig. 5D).

### Growth kinetics and changes in antibiotic susceptibility

The growth curve demonstrated no significant growth difference between the wild type and its phage-resistant mutant at the investigated time points (Fig. 5E). The antibiotic susceptibility testing for the phage-resistant mutant exhibited increased sensitivity to certain antibiotics compared to the wild-type strain. Specifically, the MICs for POL and TCY were decreased by four- and eightfold, respectively, leading to changes in their clinical interpretation, making the phage-resistant mutant intermediate and sensitive to these antibiotics, respectively. Conversely, the MICs for RIF and CAZ experienced a fourfold increase. Additionally, the MIC for AMP increased by threefold, while for CIP, it rose by twofold (Fig. 5F).

### Thermal and acid stress tolerance

**Fig 6:**
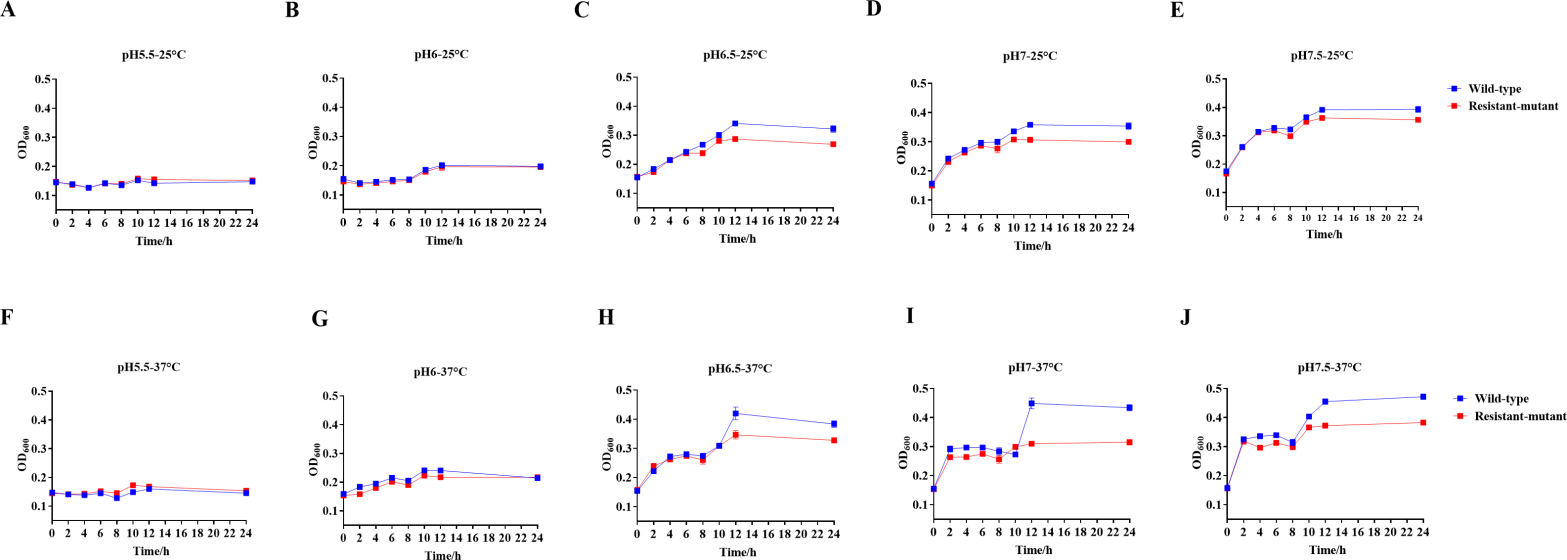
Stress tolerance of the wild type and the phage-resistant *S. dysenteriae* mutant. Bacterial growth was examined in LB broth adjusted to pH ranges of 5.5–7.5 at 25°C (**A–E**) and at 37°C (**F–J**).

The response of the wild type and its phage-resistant mutant to different environmental stressors was investigated, and OD_600_ was measured over 24 h. Notably, the phage-resistant mutant exhibited higher sensitivity than the wild-type strain to the tested pH ranges (5.5–7.5) at 25°C and 37°C. When exposed to pH 5.5, both the wild type and its phage-resistant mutant remained within the initial inoculum range after 24 h (Fig. 6A and F). At pH 6, a slight increase in growth rates was detected in either strain, with no significant differences noted in their growth after 24 h (Fig. 6B and G). Interestingly, at pH 6.5, 7, and 7.5, both strains displayed comparable growth behavior. A gradual increase was shown in their growth; however, by the end of the experiment, a slower growth of the phage-resistant mutant was evident compared to the wild-type strain, with the difference being more pronounced at 37°C (Fig. 6C, D, E, H, I, and J).

### Phage-resistant mutant had impaired biofilm formation and reduced colonization *in vivo*

**Fig 7:**
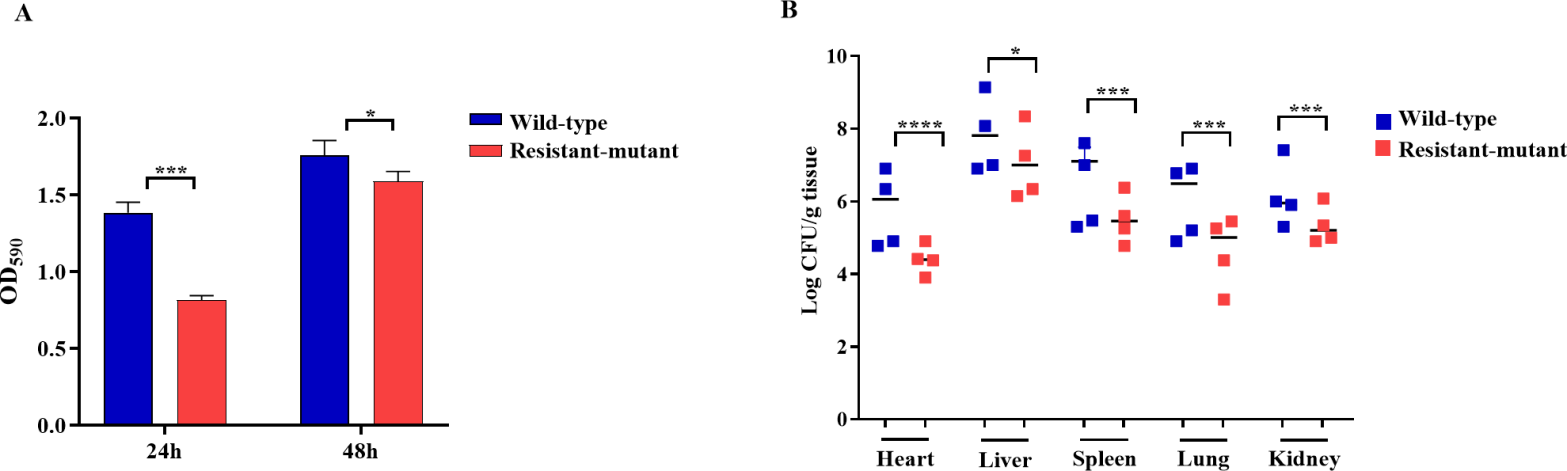
(**A**) Biofilm formation of the wild type and phage-resistant *S. dysenteriae* mutant after 24 and 48 h. (**B**) *In vivo* colonization. Bacterial load within various tissue organs was assessed after 12 h of mice infection with either the wild type or the phage-resistant mutant. *P < 0.05, ***P < 0.001, and ****P < 0.0001.

The crystal violet assay showed that the phage-resistant mutant demonstrated impaired biofilm formation compared to the wild-type strain after both 24 and 48 h, with differences being more pronounced after 24 h (Fig. 7A). Additionally, in a mouse infection model, the mean bacterial load in the heart and right kidney of mice infected with the phage-resistant mutant was decreased by approximately 2 logs compared to the wild-type strain, whereas a reduction of more than 1 log was noted in the liver, lungs, and spleen (Fig. 7B).

### Genomic analysis of phage-resistant mutant

The whole-genome resequencing of the phage-resistant mutant was conducted to explore mutations associated with phage resistance and the obtained fitness defects. A total of 19 mutations were identified in the phage-resistant mutant’s genome: 18 were insertion-deletion (indel) mutations, and one was a single nucleotide polymorphism (SNP) compared with the wild-type strain. Notably, we identified multi-base insertion in *HUZ68_RS22460* (*waaH*) gene involved in LPS biosynthesis, whereas the remaining mutations occurred in genes primarily involved in virulence, pathogenicity, stress adaptability, cellular membrane transport functions, two-component regulatory systems, transcriptional regulators, and DNA repair (Table 2).

**TABLE 2:** Identification of mutation sites in phage Henu12-resistant *S. dysenteriae* mutant through genomic resequencing

Mutation location	Mutant genes	Type of mutation	REF	ALT	Function
2065939	HUZ68_RS09935	INDEL	A	AC	The iron ABC transporter substrate-binding protein (SBP) in bacteria aids in iron acquisition and transport
2351537	HUZ68_RS11260	INDEL	ACAAATAG	A	ABC transporter permeases in bacteria mediate substrate transport and contribute to drug resistance and virulence
4017720	HUZ68_RS19145	INDEL	A	AGAGGGCAGCACAAGGTTGCGCCCATGCCACACCACTCGGCATGGGGGTCCGCTCCGGGCGATTCGAGAAGTTGAGGTTATGCGAGTCCAGGGAAGCCTAAAGC	ABC transporter SBPs in bacteria bind specific substrates for transport, essential for nutrient acquisition and pathogenicity
3323167	HUZ68_RS15895	INDEL	C	CTGGATATCGCCAACAAAACGCTACCGCTGTTTCGCCATGTCAATAGCGAGTCATTACGCAAG	Two-component regulatory proteins in bacteria sense environmental signals and modulate gene expression to adapt to stress
3173510	HUZ68_RS15200	INDEL	C	CCAGTATCACTTATTTAAGTGATAACAGATGTCTGGAAATATAGGGGCAAATCCA	YfaL aids bacterial biofilm and acid resistance
4065421	HUZ68_RS19405	INDEL	T	TTATTCCC	Members of the Ag43/Cah family function in bacterial adhesion and biofilm formation
4701757	HUZ68_RS22460	INDEL	G	GGGTAATGACTCCAACTTACTGATAGTGTTTTATGTTCAGATAATGCCCGATGACCTTGTCATGCAGCT	UDP-glucuronate:LPS(HepIII) glycosyltransferase
4035152	HUZ68_RS19240	INDEL	A	ACGCTGTAAACATTTGTCTTTAATGCACTG	Ubiquinone biosynthesis accessory factor UbiK
232715	HUZ68_RS01205	INDEL	G	GCA	Tail fiber assembly protein
396628	HUZ68_RS02035	INDEL	CT	C	RNA-guided endonuclease InsQ/TnpB family protein
465447	HUZ68_RS02345	INDEL	TA	T	Phosphorothioated DNA-binding restriction endonuclease
4726965	HUZ68_RS22595	INDEL	TGG	T	DNA damage-inducible protein D
2853727	HUZ68_RS13810	INDEL	TGCTG	T	Porin OmpC
2829312	HUZ68_RS13665	INDEL	G	GTTGACATCCTCCACGCCCTGAATGACGAGGACCCCTGCTACGTTCAGGCTGTCGCCTGAATC	SOS response-associated peptidase
2624585	HUZ68_RS12540	INDEL	T	TGTCATTGGATTTGCCCCTATATTTCCAGACATCTGTTATCACTTAAATAAGTGATACTGGTTGTCTGGAGATTCAGGGGGCCAGTCTA	NADP-specific glutamate dehydrogenase
1078264	HUZ68_RS05135	INDEL	A	ATG	Autotransporter outer membrane beta-barrel domain-containing protein
4601554	HUZ68_RS22000	INDEL	T	TGGTTGGTGCTGCCTCG	Cellulose synthase complex outer membrane protein BcsC
2230321	HUZ68_RS10725	INDEL	GGC	G	LysR family transcriptional regulator
2419505	HUZ68_RS11525	SNP	T	A	O-acetylserine/cysteine exporter

## DISCUSSION

Shigellosis, commonly known as diarrhea, is a significant global health concern (20). Among *Shigella* species, *S. dysenteriae* holds particular importance regarding this bacterial illness, exhibiting high rates of resistance to the commonly prescribed antibiotics. This complicates the successful treatment of this disease, leading to high death rates worldwide. Therefore, new effective control measures against shigellosis are urgently needed (21, 22). In the current study, a novel lytic phage, Henu12, specific for *S. dysenteriae,* was isolated from sewage samples. On double-layer agar (DLA) plates, phage Henu12 formed clear plaques surrounded by translucent halos. TEM analysis classified phage Henu12 within the *Caudoviricetes* class. Additionally, phage Henu12 demonstrated high thermal, pH, and chloroform stability. Genomic analysis showed that phage Henu12 lacks integrases, virulence, and antibiotic resistance genes. Of note, phylogenetic analysis revealed homology with phage Henu12 and other *Shigella* and *Escherichia coli* phages. This is in line with a previous report that *Shigella* species are phylogenetically embedded within various *E. coli* lineages (23). Moreover, the genomic analysis has detected the presence of 18 tRNA genes. Previous studies have shown that lytic phages encode more tRNA genes than temperate ones. Furthermore, the presence of tRNAs has been reported to facilitate phage propagation within the host and promote a more rapid and efficient translation rate (24). Collectively, the phage’s biological and genomic characteristics strengthen its promising efficacy for therapeutic application.

Recently, the combined use of phages and antibiotics has gained more attention in clinical settings. Phage Henu12 showed synergistic effects when used in combination with POL, TCY, and CAZ. This aligned with the fact that the bactericidal (POL) antibiotic interferes with the permeability and integrity of the cell membrane, along with the lysis induced by the phage. Additionally, as previously reported, phage-POL substantially perturbs bacterial metabolism, potentially resulting in antibacterial and synergistic effects (25). Besides, using sub-MIC levels of POL could help minimize its toxicity (26). With regard to the bacteriostatic (TCY) antibiotic, which inhibits bacterial protein synthesis, the obtained PAS is in line with Kamal and Dennis (27), who attributed it to the cell clustering that allows phage to move laterally across adjoined cell surfaces, thus increasing phage contact with its receptors on different cells. Moreover, a plausible explanation for the synergistic activity found between the bactericidal cell wall inhibitor (CAZ) antibiotic and phage Henu12 is that ceftazidime increases cell filamentation and the production of more phage particles (28). Altogether, these findings suggest that the PAS phenomenon is independent of the antibiotic mechanism of action, its bactericidal or bacteriostatic nature. Additionally, CIP, AMP, and RIF had additive effects; a possible explanation is that the timing and concentration of antibiotics and phages could be crucial in light of PAS rather than investigating only their simultaneous use (29).

Meanwhile, the emerging resistance against phages is a significant concern, which greatly impedes their therapeutic potential. Accordingly, a detailed understanding of the trade-offs between phage resistance and bacterial fitness is critical for phage clinical application. It has been reported that the first and potentially one of the most complicated stages of phage infection is adsorption to specific host cell receptors, including O-antigen, LPS core, outer membrane proteins, teichoic acid, capsular components, flagella, and fimbriae. Bacteria commonly develop resistance against phages by altering or losing phage-specific receptors (30, 31). If those receptors are responsible for bacterial virulence factors or antibiotic resistance mechanisms, such fitness trade-offs might attenuate bacterial virulence or restore antibiotic susceptibility in the target bacteria (32, 33). In this study, one phage-resistant *S. dysenteriae* mutant was isolated and investigated for the fitness trade-offs associated with phage resistance. The adsorption assay showed that phage Henu12 failed to adsorb to the phage-resistant mutant as opposed to the wild-type strain. This agrees with a recently reported study (34). Additionally, given that the genus *Mooglevirus*, to which phage Henu12 is proposed to belong, typically utilizes LPS as receptors, we anticipated that an alteration in the LPS profile might be the main mechanism underlying phage resistance (35). Consequently, the expression of *wzx, wzy, rfbA, waaA, waaO, waaH, lpcX, lpxM,* and* msbA* genes related to LPS biosynthesis was investigated in the wild type and its phage-resistant mutant using qRT-PCR. Interestingly, compared with the wild-type strain, the expression of the tested genes was substantially downregulated in the phage-resistant mutant, implying that LPS alterations are speculated to confer phage Henu12 resistance.

The phage-resistant mutant exhibited similar growth rates to the wild type under shaking conditions. However, phenotypic characterization of the phage-resistant mutant showed significant fitness defects, including increased susceptibility to certain antibiotics (TCY and POL) along with reduced biofilm formation when compared to the wild-type strain. Given that *S. dysenteriae* infections occur within mammalian hosts, stress tolerance assays were conducted at physiologically relevant conditions that are representative of *S. dysenteriae*’s infection environment. The phage-resistant mutant exhibited higher sensitivity to the investigated pH and temperature ranges than the wild-type strain. Therefore, the altered tolerance in the phage-resistant mutant to these stressors might influence *Shigella* transmission, virulence, and survival, complementing the classical fitness assessments (36). Of note, unlike the earlier growth curve findings, the stress tolerance assay demonstrated slower growth rates of the phage-resistant mutant compared to the wild type, particularly within certain tested pH ranges, under non-shaking conditions. These findings indicate that the fitness cost associated with phage resistance becomes evident under oxygen-limited or nutrient-gradient conditions (37, 38). It is intriguing to note that the phage-resistant mutant demonstrated diminished colonization *in vivo* compared to the wild-type strain. Consequently, phage resistance is surmised to induce a fitness cost, rendering the phage-resistant mutant more vulnerable to host-mediated clearance by the immune system than the wild-type strain (39).

Additionally, we postulated that our phage-resistant mutant likely harbors mutations in its genome that confer phage resistance and fitness defects. Comparative genomic analysis showed 18 indels and 1 SNP within the phage-resistant mutant genome (Table 2). Consistent with our earlier RT-PCR results, we identified a mutation in the *waaH* gene, which encodes for an enzyme that modifies LPS by catalyzing the addition of glucuronic acid to heptose III of the inner core oligosaccharide. A mutation in this gene is predicted to truncate this enzyme, likely altering the LPS core structure. Given that our phage represents a new species, it may possess distinct receptor requirements or sensitivity to LPS modifications. Therefore, the disruptions in *waaH* genes (downregulation or mutation) could prevent phage Henu12 adsorption, presumably explaining the phage-resistant phenotype. Similarly, disruptions in *waa* genes have been previously reported to influence phage infectivity (40).

It has been reported that the deletion of *DppA1*, an ABC membrane transporter protein and substrate-binding protein encoded by *Pseudomonas aeruginosa,* decreases biofilm formation (41). Several studies have also shown that ABC transporter proteins play critical roles in the virulence and pathogenesis of major pathogenic species, including *Streptococcus pneumoniae* and *E. coli* (42, 43). Moreover, several two-component systems are involved in host cell invasion, proliferation inside the host, survival in environmental stresses, and immune evasion (44). The PhoQ/PhoP, OmpR/EnvZ, CpxA/CpxR, EvgS/EvgA, and ArcB/ArcA systems have been reported to contribute to *Shigella* virulence and intracellular colonization (45). Studies have reported that *Salmonella*’s tolerance to stressful environments is linked to various two-component regulatory systems. The PhoP/PhoQ system aids in self-adaptation to acidic stress (46), PmrA/PmrB enhances resistance to host antimicrobial peptides (47), and OmpR/EnvZ improves tolerance to osmotic stress (48, 49).

Autotransporter proteins have been identified in a diverse array of gram-negative bacteria and are often linked to virulence functions, including adhesion, auto-agglutination, biofilm formation, and other virulence-related activities. For instance, the glycosylated adhesin AIDA-1, a type Va autotransporter protein in *E. coli*, binds to the epithelial cells of various animal species and is a significant virulence factor responsible for causing diarrhea in pigs (50). Meanwhile, the deletion of the Adh adhesin domain of the trimeric auto-transporter Apa1 in *Actinobacillus pleuropneumoniae* impacted the bacteria’s cell adhesion, auto-agglutination, biofilm formation, and pathogenicity (51). In addition, UbiK, an accessory factor, has been reported to be required for *in vivo* virulence in *Salmonella enterica* (52). Moreover, the transcriptional regulator (LysR) family proteins control genes involved in virulence and quorum sensing (53). Altogether, the identified mutations are presumably related to the identified fitness trade-offs associated with phage resistance.

In conclusion, the present study demonstrated comprehensive biological and genomic features of a novel lytic *S. dysenteriae* phage, Henu12. Synergy was noted when phage Henu12 was used in combination with polymyxin B, tetracycline, and ceftazidime. The isolated phage-resistant mutant incurred fitness costs as a trade-off. Comparative genomic analysis identified a mutation in the LPS biosynthesis (*waaH*) gene, which is likely the primary reason for the phage-resistance mechanism, along with other mutations that could be responsible for the associated fitness defects. Understanding the fitness trade-offs might fill some gaps in the future success of phage therapy in combating resistant shigellosis. Further evidence from clinical studies is also warranted, as emphasized by the World Health Organization, before phages are widely available for human use to ensure their efficacy, safety, and viability in all sectors of One Health.

## MATERIALS AND METHODS

### Phage isolation, purification, and propagation

Sewage water samples were collected from a poultry farm for phage isolation as previously described (54). In brief, a 30 mL wastewater sample was centrifuged, and the supernatant was filtered using a 0.22 µm filter. Subsequently, an aliquot of 2 mL of *S. dysenteriae* serotype O2 culture was added to the supernatant, followed by incubation for 24 h at 37°C with shaking. The presence of phages was detected using the spot assay (55). Phage purification was performed using the double-layer agar technique (56). The purified phage was propagated in liquid culture by coculturing the phage with logarithmic-phase *S. dysenteriae* for 6 h at 37°C with shaking before being centrifuged. The supernatant was filtered, and then the phage titer was evaluated using the DLA technique (57).

### Transmission electron microscopy

Ten microliters of purified phage (10^10^ PFU/mL) was placed on a copper grid and negatively stained with phosphotungstic acid solution (2%). The grid was then visualized under the FEI Tecnai G2 Spirit 120 kV TEM (58).

### Multiplicity of infection and one-step growth curve

Logarithmic-phase *S. dysenteriae* (1 × 10^8^ CFU/mL) was cocultured with phage Henu12 at different MOIs (0.0001, 0.001, 0.01, 0.1, 1, and 10), followed by incubation for 6 h at 37°C with shaking. The titers of the phage of different MOIs were determined using the DLA technique (59). For the one-step growth curve, at the optimal MOI, the phage Henu12 was mixed with logarithmic-phase *S. dysenteriae* and incubated at 37°C with shaking. Samples were taken every 20 min for phage titration (60). The latent period and burst size of phage Henu12 were determined.

### Stability studies

The phage stability was assessed as previously described (61, 62). Briefly, phage Henu12 was incubated at temperatures ranging from 4°C to 70°C, different pH values (pH 3–12), and chloroform at ratios of 20%, 40%, 60%, 80%, and 100% for 1 h. Then, the phage titer was assessed using the DLA technique. The stability of phage Henu12 under ultraviolet light was assessed as previously described (63), and the phage titer was determined after 0, 5, 10, 30, 60, 90, and 120 min.

### Genome sequencing and bioinformatics analysis

The phage DNA was extracted as previously described (64). The whole-genome sequencing of the extracted phage genome was performed by Shanghai Sangong Biological Co., Ltd. using the Illumina NovaSeq 6000 platform. Homologous DNA and protein sequence alignments were performed using the Basic Local Alignment Search Tool (BLAST) for nucleotide (BLASTN) and for proteins (BLASTP). Prokka was employed to predict the open reading frames and annotate the potential functions of predicted ORFs (65). Proksee (https://proksee.ca/) was employed to map the Henu12 phage genome. tRNAscan-SE was used to detect tRNA in the phage genome (http://lowelab.ucsc.edu/tRNAscan-SE/). TaxMyPhage (https://ptax.ku.dk/) was used for phage taxonomic classification. The antibiotic resistance genes, integrases, and the virulence factors were predicted using the comprehensive antibiotic resistance database Resistance Gene Identifier tool (https://card.mcmaster.ca/analyze/rgi), PHASTER (https://phaster.ca/), and the Virulence Factors Database (VFDB) (https://www.mgc.ac.cn/VFs/), respectively.

The phylogenetic analysis was constructed by MEGA11 software (66). First, the complete genome sequence of phage Henu12 was imported into the NCBI BLASTN platform (https://blast.ncbi.nlm.nih.gov/) to check for similarity with the other reported phage genomes. Subsequently, the whole-genome sequences of phages with higher homology were downloaded from GenBank, sequences were aligned using ClustalW, and the phylogenetic tree was constructed using the neighbor-joining method and 1,000 bootstrap replicates. The evolutionary distances were computed using the Maximum Composite Likelihood method (67). In addition, the phylogenetic relationship based on the amino acid sequences of the terminase large subunit was conducted (66). The comparison of the phage genome with closely related phages (*Escherichia* phage vB_EcoM_3HA14 and *Shigella* phage Sf15) was accomplished using Easyfig (68).

### Phage-antibiotic synergy assay

The microbroth dilution method was first employed to assess the minimum inhibitory concentration values of six different antibiotics, including polymyxin B, tetracycline, ceftazidime, ciprofloxacin, ampicillin, and rifampicin (69). A time-kill assay was conducted to explore the potential phage-antibiotic synergy (PAS) by using sub-MIC values of the tested antibiotics. Samples were incubated at 37°C with shaking and subjected to viable counts every 1 h over 8 h (70). The interaction plots were analyzed using a two-way ANOVA to investigate the possible PAS.

### Isolation of phage-resistant *S. dysenteriae* mutant

A logarithmic phase culture of *S. dysenteriae* host strain was infected with phage Henu12 (10^10^ PFU/mL) and incubated for 48 h, followed by plating on LB plates. Emerging colonies were randomly selected and subsequently streaked twice on LB plates for single colony isolation (71). The frequency of phage-resistant mutants was determined as previously described, and the frequency of mutants was calculated by dividing the number of surviving colonies by the total number of sensitive bacteria (72). Confirmation of phage resistance was further performed using the spot assays and the inverted spot assays.

### Adsorption assay

The adsorption of phage Henu12 onto the wild type and its phage-resistant mutant was conducted at an MOI of 0.01, as previously described (39). The initial concentrations of the tested bacterial strains and phage Henu12 were 1 × 10^8^ CFU/mL and 8 × 10^6^ PFU/mL, respectively. Subsequently, the phage-bacteria co-cultures were incubated at 37°C. At 0, 5, 10, and 15 min, samples were centrifuged and filtered using a 0.22 µm filter to assess free phage titers. The adsorption rate constant for phage Henu12 was calculated using the equation below (73).


$$k=-ln\left(\frac{P}{P0}\right)/Bt,$$

*k* is the adsorption rate constant (mL/min), *P* is the free phage concentration per mL, *P*_0_ is the initial phage concentration, *B* is the initial bacterial density, and *t* is the time (min).

### Quantitative reverse transcription PCR

To inspect the role of lipopolysaccharide in phage-host interaction and resistance mechanism, the expression levels of nine genes involved in LPS biosynthesis, *wzx, wzy, rfbA, waaA, waaO, waaH, lpcX, lpxM,* and *msbA*, in *S. dysenteriae* were assessed by qRT-PCR. Briefly, logarithmic-phase LB broth cultures of the wild type and its phage-resistant mutant were subjected to total RNA extraction using the Coolaber Bacterial RNA Extraction Kit (Coolaber, Beijing, China) following the manufacturer’s instructions. cDNA synthesis was performed using HiScript III RT SuperMix for qPCR (Vazyme, Nanjing, China) following the manufacturer’s instructions. Amplification was carried out with Servicebio 2× Universal Blue SYBR Green qPCR Master Mix (Servicebio, Wuhan, China), with the primers listed in Table S1. Primers were custom-designed according to the target sequences obtained from FASTA files by Wuhan ServiceBio Biotechnology Co., Ltd. The values were normalized to *16s rRNA*. The 2^-△△Ct^ method was employed to calculate the gene expression levels.

### Growth curve and antimicrobial susceptibility testing

The *in vitro* growth curves of either the wild type or its phage-resistant mutant were assessed by growing the bacterial cultures in LB broth at 37°C with shaking, and the optical density (OD_600_) was measured over 15 h (16). The MIC values of the six antibiotics previously mentioned were re-evaluated against the phage-resistant mutant (69).

### Stress tolerance assays

For stress tolerance assay, stationary-phase cultures of the wild type and its phage-resistant mutant were grown in fresh LB broth adjusted to pH ranges (5.5–7.5) and incubated at 25°C and 37°C under static conditions. The bacterial response to environmental stressors was determined by measuring OD_600_ at 0, 2, 4, 6, 8, 10, 12, and 24 h (74).

### Biofilm formation assay

The biofilm-forming capability of the wild type and its phage-resistant mutant was investigated using the crystal violet staining. Briefly, overnight cultures of the tested strains were transferred into 96-well microtiter plates and then incubated for 24 and 48 h at 37°C under static conditions. After that, the content of the wells was gently removed and washed with phosphate-buffered saline (PBS). The total biofilm biomass was determined by measuring OD at 590 nm (75).

### Mouse infection model

The mouse infection model was utilized to evaluate the *in vivo* colonization ability of the wild type and its phage-resistant mutant, as previously described with some modifications (39). Mice were divided into two groups, each comprising four female BALB/c mice 6–10 weeks old. Suspensions of either the wild type or its phage-resistant mutant at a concentration of 10^8^ CFU were administered to the mice via intraperitoneal injection. After 12 h of infection, mice were euthanized, and tissues, including the lungs, liver, right kidney, heart, and spleen, were collected, rinsed, weighed, and homogenized in PBS for CFU quantification.

### DNA extraction, library preparation, and next-generation sequencing

For whole-genome resequencing of the phage-resistant mutant, genomic DNA (gDNA) was extracted using the Coolaber Bacterial DNA Extraction Kit (Coolaber, Beijing, China) according to the manufacturer’s instructions, quantified using the Qubit dsDNA HS assay kit (Sangon, Shanghai, China), and subjected to 1% agarose gel electrophoresis to confirm integrity. The DNA library generation and next-generation sequencing were performed by Sangon Biotech Co., Ltd. (Shanghai, China) according to the manufacturer’s instructions. First, 500 ng of quantified gDNA was fragmented by Covaris (Woburn, USA), then the Hieff NGS MaxUp II DNA Library Prep Kit for Illumina (YEASEN, Shanghai, China) was used for the next steps. Briefly, the Endprep enzyme was added to repair the end and 3′ end A-tail ligation. Then, the adaptor was ligated by the enhancer and Fast T4 DNA ligase. An index primer was added by PCR, and the amplified product of about 400 bp was selected by DNA selection beads. The gDNA library’s final concentration and size were confirmed by Qubit 4.0 (Thermo, Waltham, MA, USA) and 2% agarose gel electrophoresis, respectively. The libraries were pooled and loaded on a NovaSeq 6000 (Illumina, San Diego, USA)/DNBseq-T7 (BGI, Shenzhen, China) sequencer by 2 × 150 bp paired-end sequence kit according to the manufacturer’s instructions. *S. dysenteriae* SWHEFF_49 was used as a reference strain for comparative genome sequencing.

### Statistical analysis

Statistical analyses were conducted using GraphPad Prism 10.1 software, primarily utilizing the *t*-test or one-way ANOVA unless otherwise indicated. Data represent the mean ± standard deviation of three independent experiments.

## Data Availability

Complete genome of *Shigella dysenteriae* phage Henu12 is available in https://www. ncbi. nlm.nih.gov/ nuccore/2533208574 under the GenBank accession number OQ834936.1.

## SUPPLEMENTAL MATERIAL

Supplemental tables spectrum.01150-25-s0001.docx Tables S1 to S4.

ASM does not own the copyrights to Supplemental Material that may be linked to, or accessed through, an article. The authors have granted ASM a non-exclusive, world-wide license to publish the Supplemental Material files. Please contact the corresponding author directly for reuse.
