# Supplementary material for: Phage Henu12-resistant mutant derives fitness trade-offs in Shigella dysenteriae
Source: Microbiol Spectr. 2025 Oct 27;13(12):e01150-25. doi: 10.1128/spectrum.01150-25 (PMC12671091; doi:10.1128/spectrum.01150-25)
Supplement: Supplemental tables — Tables S1 to S4. [file spectrum.01150-25-s0001.docx]

**Supplementary Table S1:** Primers for qRT-PCR

| **Genes** | **Primers** | **Sequence (5'-3')** |
| --- | --- | --- |
| *wzx* | wzx-F | CAAGTCGTGCGATCTCACAGA |
|  | wzx-R | AGTAAACACGCGGCGCATC |
| *wzy* | wzy-F | TTGCTCCAGAAGTGAGG |
|  | wzy-R | GTGGTGGTGGAAGATTAC |
| *rfbA* | rfbA-F | TGATGGACCCGGAACGCT |
|  | rfbA-R | TGTCGTAGAAATAAAGCCCGGTC |
| *waaA* | waaA-F | TCAACCGTTTCCTGAATAAAGTCG |
|  | waaA-R | CGACGGACGAATTTACCCA |
| *waaO* | waaO-F | GGTCTATTGCCATGTATTTCCGTT |
|  | waaO-R | CGTTCAGTAACAACAGCAGCAA |
| *waaH* | waaH-F | ATCGTGGGATTGAAGTGGCA |
|  | waaH-R | CCAGGTTTCTCCCGTTTCA |
| *lpcX* | lpcX-F | CCGTTTGTATACCTGCTGCTTGA |
|  | lpcX-R | CAGCCCACTTATCGCCATCTT |
| *lpxM* | lpxM-F | TCAATCTGTCGCTCTGCTTTCC |
|  | lpxM-R | TTCTCGTTATTACGCCGCATCT |
| *msbA* | msbA-F | CCACTTCTTGATGATGGCTTTG |
|  | msbA-R | GCAACCTGTTCGGAATCGTAG |
| *16s rRNA* | 16s rRNA-F | TGGAGCATGTGGTTTAATTCGA |
|  | 16s rRNA-R | TGCGGGACTTAACCCAACA |

**Supplementary Table S2:** Detailed genome annotation of phage Henu12

| **ORF** | **Start** | **Stop** | **Amino acids** | **Strand** | **Molecular weight in KDa** | **Product** | **DbXrefs** |
| --- | --- | --- | --- | --- | --- | --- | --- |
| ORF1 | 320 | 700 | 126 | - | 14.4 | hypothetical protein | NCBI_GP:WKB10154.1 |
| ORF2 | 959 | 1165 | 68 | - | 8.5 | hypothetical protein | NCBI_GP:WKB10155.1 |
| ORF3 | 1388 | 1801 | 137 | + | 15.8 | hypothetical protein | NCBI_GP:WKB10136.1 |
| ORF4 | 1670 | 2521 | 283 | - | 31.5 | hypothetical protein | NCBI_GP:WKB10156.1 |
| ORF5 | 2659 | 2880 | 73 | - | 8.5 | hypothetical protein | NCBI_GP:WKB10157.1 |
| ORF6 | 2855 | 3181 | 108 | - | 12.4 | hypothetical protein | NCBI_GP:WKB10158.1 |
| ORF7 | 3338 | 3607 | 89 | - | 10.6 | hypothetical protein | NCBI_GP:WKB10159.1 |
| ORF8 | 3604 | 3873 | 89 | - | 10.1 | o-spanin | NCBI_GP:WKB10160.1 |
| ORF9 | 3767 | 4114 | 115 | - | 13.2 | putative i-spanin | NCBI_GP:WKB10161.1 |
| ORF10 | 4167 | 4631 | 154 | - | 16.3 | hypothetical protein | NCBI_GP:WKB10162.1 |
| ORF11 | 4643 | 5338 | 231 | - | 26.1 | polynucleotide kinase | NCBI_GP:WKB10163.1 |
| ORF12 | 5316 | 5888 | 190 | - | 21.7 | hypothetical protein | NCBI_GP:WKB10164.1 |
| ORF13 | 5966 | 7087 | 373 | - | 41.7 | putative rIIB protein | NCBI_GP:WKB10165.1 |
| ORF14 | 7168 | 9540 | 790 | - | 90.4 | putative rIIA protein | NCBI_GP:WKB10166.1 |
| ORF15 | 9764 | 10099 | 111 | - | 12.9 | hypothetical protein | NCBI_GP:WKB10167.1 |
| ORF16 | 10153 | 11979 | 608 | - | 68.4 | nicotinamide phosphoribosyl transferase | NCBI_GP:WKB10224.1 |
| ORF17 | 12024 | 12905 | 293 | - | 32.3 | ribose-phosphate pyrophosphokinase | NCBI_GP:WKB10168.1 |
| ORF18 | 12916 | 13209 | 97 | - | 11.0 | hypothetical protein | NCBI_GP:WKB10169.1 |
| ORF19 | 13187 | 13702 | 171 | - | 20.2 | hypothetical protein | NCBI_GP:WKB10170.1 |
| ORF20 | 13755 | 14066 | 103 | - | 11.9 | putative HNH endonuclease | NCBI_GP:WKB10171.1 |
| ORF21 | 14078 | 14335 | 85 | - | 8.9 | hypothetical protein | NCBI_GP:WKB10172.1 |
| ORF22 | 14351 | 14749 | 132 | - | 14.8 | hypothetical protein | NCBI_GP:WKB10173.1 |
| ORF23 | 14634 | 15119 | 161 | - | 18.7 | anaerobic NTP reductase, small subunit | NCBI_GP:WKB10174.1 |
| ORF24 | 15119 | 15511 | 130 | - | 15.0 | putative DNA methylase | NCBI_GP:WKB10175.1 |
| ORF25 | 15608 | 15865 | 85 | - | 10.3 | hypothetical protein | NCBI_GP:WKB10225.1 |
| ORF26 | 16109 | 18250 | 713 | - | 80.6 | anaerobic NTP reductase, large subunit | NCBI_GP:WKB10176.1 |
| ORF27 | 18493 | 18735 | 80 | - | 9.0 | glutaredoxin | NCBI_GP:WKB10177.1 |
| ORF28 | 18735 | 19808 | 357 | - | 41.3 | ribonucleotide triphosphate reductase, small subunit | NCBI_GP:WKB10178.1 |
| ORF29 | 19805 | 20164 | 119 | - | 14.1 | hypothetical protein | NCBI_GP:WKB10179.1 |
| ORF30 | 20136 | 22370 | 744 | - | 85.2 | ribonucleoside-diphosphate reductase subunit  alpha | NCBI_GP:WKB10180.1 |
| ORF31 | 22422 | 22757 | 111 | - | 12.8 | hypothetical protein | NCBI_GP:WKB10181.1 |
| ORF32 | 22750 | 23076 | 108 | - | 12.6 | hypothetical protein | NCBI_GP:WKB10182.1 |
| ORF33 | 23057 | 23812 | 251 | - | 28.7 | hypothetical protein | NCBI_GP:WKB10183.1 |
| ORF34 | 23812 | 24042 | 76 | - | 8.8 | hypothetical protein | NCBI_GP:WKB10184.1 |
| ORF35 | 24029 | 24529 | 166 | - | 19.5 | GIY-YIG homing endonuclease | NCBI_GP:WKB10185.1 |
| ORF36 | 24526 | 25062 | 178 | - | 20.6 | HNH endonuclease | NCBI_GP:WKB10186.1 |
| ORF37 | 25004 | 26077 | 357 | - | 40.7 | putative exodeoxyribonuclease | NCBI_GP:WKB10187.1 |
| ORF38 | 26113 | 26973 | 286 | - | 31.9 | hypothetical protein | NCBI_GP:WKB10188.1 |
| ORF39 | 27193 | 27474 | 93 | - | 11.0 | hypothetical protein | NCBI_GP:WKB10189.1 |
| ORF40 | 27446 | 29437 | 663 | - | 74.9 | DNA primase/helicase | NCBI_GP:WKB10190.1 |
| ORF41 | 29634 | 30374 | 246 | - | 28.0 | hypothetical protein | NCBI_GP:WKB10191.1 |
| ORF42 | 30431 | 31225 | 264 | - | 29.3 | hypothetical protein | NCBI_GP:WKB10192.1 |
| ORF43 | 31209 | 31649 | 146 | - | 173 | hypothetical protein | NCBI_GP:WKB10193.1 |
| ORF44 | 31909 | 34608 | 899 | + | 103.3 | DNA polymerase | NCBI_GP:WKB10137.1 |
| ORF45 | 34608 | 35120 | 170 | + | 19.4 | HNH endonuclease motif protein | NCBI_GP:WKB10138.1 |
| ORF46 | 35172 | 35390 | 72 | + | 8.1 | hypothetical protein | NCBI_GP:WKB10139.1 |
| ORF47 | 35387 | 35596 | 69 | + | 8.1 | hypothetical protein | NCBI_GP:WKB10140.1 |
| ORF48 | 35684 | 35914 | 76 | + | 9.3 | hypothetical protein | NCBI_GP:WKB10141.1 |
| ORF49 | 36166 | 36402 | 78 | + | 8.7 | hypothetical protein | NCBI_GP:WKB10142.1 |
| ORF50 | 36362 | 36619 | 85 | + | 10.2 | hypothetical protein | NCBI_GP:WKB10143.1 |
| ORF51 | 36711 | 37811 | 366 | + | 42.5 | DNA ligase | NCBI_GP:WKB10144.1 |
| ORF52 | 38053 | 38433 | 126 | + | 142 | hypothetical protein | NCBI_GP:WKB10145.1 |
| ORF53 | 38436 | 38639 | 67 | + | 7.7 | hypothetical protein | NCBI_GP:WKB10146.1 |
| ORF54 | 38632 | 38931 | 99 | + | 11.5 | transcriptional regulatory protein | NCBI_GP:WKB10147.1 |
| ORF55 | 38933 | 39292 | 119 | + | 13.4 | hypothetical protein | NCBI_GP:WKB10148.1 |
| ORF56 | 39277 | 39780 | 167 | + | 19.6 | hypothetical protein | NCBI_GP:WKB10149.1 |
| ORF57 | 39782 | 40297 | 171 | + | 19.5 | hypothetical protein | NCBI_GP:WKB10150.1 |
| ORF58 | 40294 | 40566 | 90 | + | 10.1 | hypothetical protein | NCBI_GP:WKB10151.1 |
| ORF59 | 40563 | 41108 | 181 | + | 20.3 | dihydrofolate reductase | NCBI_GP:WKB10152.1 |
| ORF60 | 41110 | 42009 | 299 | + | 34.3 | thymidylate synthase | NCBI_GP:WKB10153.1 |
| ORF61 | 42046 | 42420 | 124 | - | 13.8 | putative holin | NCBI_GP:WKB10194.1 |
| ORF62 | 42701 | 45232 | 843 | - | 89.9 | tail fiber protein | NCBI_GP:WKB10195.1 |
| ORF63 | 45278 | 46486 | 402 | - | 42.6 | putative tail fiber protein | NCBI_GP:WKB10196.1 |
| ORF64 | 46489 | 46788 | 99 | - | 10.9 | hypothetical protein | NCBI_GP:WKB10197.1 |
| ORF65 | 46788 | 47645 | 285 | - | 31.5 | hypothetical protein | NCBI_GP:WKB10198.1 |
| ORF66 | 47648 | 49117 | 489 | - | 53.3 | baseplate assembly protein | NCBI_GP:WKB10199.1 |
| ORF67 | 49117 | 49533 | 138 | - | 15.5 | hypothetical protein | NCBI_GP:WKB10200.1 |
| ORF68 | 49533 | 50174 | 213 | - | 23.7 | hypothetical protein | NCBI_GP:WKB10201.1 |
| ORF69 | 50171 | 51148 | 325 | - | 37.1 | hypothetical protein | NCBI_GP:WKB10202.1 |
| ORF70 | 51148 | 51489 | 113 | - | 12.9 | hypothetical protein | NCBI_GP:WKB10203.1 |
| ORF71 | 51489 | 52295 | 268 | - | 28.9 | hypothetical protein | NCBI_GP:WKB10204.1 |
| ORF72 | 52295 | 54532 | 745 | - | 81.0 | tail tape measure protein | NCBI_GP:WKB10205.1 |
| ORF73 | 54532 | 54759 | 75 | - | 8.6 | putative tail tape measure chaperone | NCBI_GP:WKB10206.1 |
| ORF74 | 54768 | 55169 | 133 | - | 14.7 | putative tail tape measure chaperone | NCBI_GP:WKB10207.1 |
| ORF75 | 55246 | 55692 | 148 | - | 16.2 | hypothetical protein | NCBI_GP:WKB10208.1 |
| ORF76 | 55707 | 57059 | 450 | - | 48.9 | hypothetical protein | NCBI_GP:WKB10209.1 |
| ORF77 | 57060 | 57659 | 199 | - | 22.3 | hypothetical protein | NCBI_GP:WKB10210.1 |
| ORF78 | 57634 | 58035 | 133 | - | 15.5 | hypothetical protein | NCBI_GP:WKB10211.1 |
| ORF79 | 58032 | 58514 | 160 | - | 17.7 | hypothetical protein | NCBI_GP:WKB10212.1 |
| ORF80 | 58514 | 58963 | 149 | - | 17.0 | hypothetical protein | NCBI_GP:WKB10213.1 |
| ORF81 | 58986 | 60089 | 367 | - | 41.5 | major capsid protein | NCBI_GP:WKB10214.1 |
| ORF82 | 60121 | 60498 | 125 | - | 13.5 | hypothetical protein | NCBI_GP:WKB10215.1 |
| ORF83 | 60511 | 61857 | 448 | - | 48.2 | head maturation protease | NCBI_GP:WKB10216.1 |
| ORF84 | 61869 | 62201 | 110 | - | 11.6 | hypothetical protein | NCBI_GP:WKB10217.1 |
| ORF85 | 62201 | 62701 | 166 | - | 18.4 | hypothetical protein | NCBI_GP:WKB10218.1 |
| ORF86 | 62701 | 64167 | 488 | - | 55.3 | hypothetical protein | NCBI_GP:WKB10219.1 |
| ORF87 | 64180 | 65781 | 533 | - | 60.0 | terminase large subunit | NCBI_GP:WKB10220.1 |
| ORF88 | 65803 | 66009 | 68 | - | 7.7 | hypothetical protein | NCBI_GP:WKB10221.1 |
| ORF89 | 66404 | 67165 | 253 | - | 26.6 | hypothetical protein | NCBI_GP:WKB10222.1 |
| ORF90 | 67619 | 67831 | 70 | - | 8.3 | hypothetical protein | NCBI_GP:WKB10223.1 |

**Supplementary Table S3**: Details of tRNAs predicted in Henu12 genome

| **tRNA No.** | **Location (bp)** | **Length (bp)** | **Amino acid** | **Anticodon** |
| --- | --- | --- | --- | --- |
| 1 | 66227-66302 | 76 | Cys | GCA |
| 2 | 66310-66402 | 93 | Ser | GTA |
| 3 | 67155-67230 | 76 | Phe | GAA |
| 4 | 67238-67314 | 77 | His | GTC |
| 5 | 67346-67421 | 76 | Gln | CTG |
| 6 | 67428-67506 | 79 | Leu | TAA |
| 7 | 67509-67583 | 75 | Gln | GAA |
| 8 | 67593-67669 | 77 | Arg | ACG |
| 9 | 67847-67924 | 78 | Leu | TAA |
| 10 | 67926-68000 | 75 | Val | TAC |
| 11 | 68097-68173 | 77 | Thr | TGT |
| 12 | 68180-68254 | 75 | Gly | TCC |
| 13 | 68261-68336 | 76 | Ala | TGC |
| 14 | 68343-68418 | 76 | Lys | CTT |
| 15 | 68426-68502 | 77 | Leu | TAG |
| 16 | 68754-68841 | 88 | Ser | GAA |
| 17 | 69079-69155 | 77 | Arg | TCT |
| 18 | 69432-69507 | 76 | Ile | GAT |

| **Phage** | **Initial phage concentration (P0)** | **Free phage concentration (P)** | **Adsorption rate constant (k)** | **% of adsorbed phages** |
| --- | --- | --- | --- | --- |
| Henu5 | 8000000/mL | 300000/mL | 2.19 × 10^-9^ mL/min | ~96% |

**Table S4:** Phage adsorption assay
